# Supplementary material for: Resolving the full spectrum of human genome variation using Linked-Reads
Source: Genome Res. 2019 Apr;29(4):635–45. doi: 10.1101/gr.234443.118 (PMC6442396; doi:10.1101/gr.234443.118)
Supplement: Supplemental Material [file supp_gr.234443.118_Supplemental_longranger-2.2.zip › longranger-2.2.tar.bz2!/lr2.2-public/lib/stan/cmdstan/stan/doc/api/html/var__context_8hpp_source.html]

Stan: src/stan/io/var\_context.hpp Source File


|  |  |
| --- | --- |
|  | Stan  2.5.0  probability, sampling & optimization |


- Main Page
- Related Pages
- Namespaces
- Classes
- Files

- File List
- File Members

All Classes Namespaces Files Functions Variables Typedefs Enumerations Enumerator Friends Macros Pages

- src
- stan
- io

var\_context.hpp

Go to the documentation of this file.

1 #ifndef STAN\_\_IO\_\_VAR\_CONTEXT\_HPP

2 #define STAN\_\_IO\_\_VAR\_CONTEXT\_HPP

3

4 #include <sstream>

5 #include <stdexcept>

6 #include <string>

7 #include <vector>

8

9 namespace stan {

10

11  namespace io {

12

29  class var\_context {

30  public:

31  virtual ~var\_context() {};

32

42  virtual bool contains\_r(const std::string& name) const = 0;

43

56  virtual std::vector<double> vals\_r(const std::string& name) const = 0;

57

66  virtual std::vector<size\_t> dims\_r(const std::string& name) const = 0;

67

76  virtual bool contains\_i(const std::string& name) const = 0;

77

86  virtual std::vector<int> vals\_i(const std::string& name) const = 0;

87

96  virtual std::vector<size\_t> dims\_i(const std::string& name) const = 0;

97

104  virtual void names\_r(std::vector<std::string>& names) const = 0;

105

112  virtual void names\_i(std::vector<std::string>& names) const = 0;

113

114  void add\_vec(std::stringstream& msg,

115  const std::vector<size\_t>& dims) const {

116  msg << '(';

117  for (size\_t i = 0; i < dims.size(); ++i) {

118  if (i > 0) msg << ',';

119  msg << dims[i];

120  }

121  msg << ')';

122  }

123

124  void validate\_dims(const std::string& stage,

125  const std::string& name,

126  const std::string& base\_type,

127  const std::vector<size\_t>& dims\_declared) const {

128  bool is\_int\_type = base\_type == "int";

129  if (is\_int\_type) {

130  if (!contains\_i(name)) {

131  std::stringstream msg;

132  msg << (contains\_r(name)

133  ? "int variable contained non-int values"

134  : "variable does not exist" )

135  << "; processing stage=" << stage

136  << "; variable name=" << name

137  << "; base type=" << base\_type;

138  throw std::runtime\_error(msg.str());

139  }

140  } else {

141  if (!contains\_r(name)) {

142  std::stringstream msg;

143  msg << "variable does not exist"

144  << "; processing stage=" << stage

145  << "; variable name=" << name

146  << "; base type=" << base\_type;

147  throw std::runtime\_error(msg.str());

148  }

149  }

150  std::vector<size\_t> dims = dims\_r(name);

151  if (dims.size() != dims\_declared.size()) {

152  std::stringstream msg;

153  msg << "mismatch in number dimensions declared and found in context"

154  << "; processing stage=" << stage

155  << "; variable name=" << name

156  << "; dims declared=";

157  add\_vec(msg,dims\_declared);

158  msg << "; dims found=";

159  add\_vec(msg,dims);

160  throw std::runtime\_error(msg.str());

161  }

162  for (size\_t i = 0; i < dims.size(); ++i) {

163  if (dims\_declared[i] != dims[i]) {

164  std::stringstream msg;

165  msg << "mismatch in dimension declared and found in context"

166  << "; processing stage=" << stage

167  << "; variable name=" << name

168  << "; position="

169  << i

170  << "; dims declared=";

171  add\_vec(msg,dims\_declared);

172  msg << "; dims found=";

173  add\_vec(msg,dims);

174  throw std::runtime\_error(msg.str());

175  }

176  }

177  }

178

179  static std::vector<size\_t> to\_vec() {

180  return std::vector<size\_t>();

181  }

182  static std::vector<size\_t> to\_vec(size\_t n1) {

183  std::vector<size\_t> v(1);

184  v[0] = n1;

185  return v;

186  }

187  static std::vector<size\_t> to\_vec(size\_t n1, size\_t n2) {

188  std::vector<size\_t> v(2);

189  v[0] = n1;

190  v[1] = n2;

191  return v;

192  }

193  static std::vector<size\_t> to\_vec(size\_t n1, size\_t n2,

194  size\_t n3) {

195  std::vector<size\_t> v(3);

196  v[0] = n1;

197  v[1] = n2;

198  v[2] = n3;

199  return v;

200  }

201  static std::vector<size\_t> to\_vec(size\_t n1, size\_t n2,

202  size\_t n3, size\_t n4) {

203  std::vector<size\_t> v(4);

204  v[0] = n1;

205  v[1] = n2;

206  v[2] = n3;

207  v[3] = n4;

208  return v;

209  }

210  static std::vector<size\_t> to\_vec(size\_t n1, size\_t n2,

211  size\_t n3, size\_t n4,

212  size\_t n5) {

213  std::vector<size\_t> v(5);

214  v[0] = n1;

215  v[1] = n2;

216  v[2] = n3;

217  v[3] = n4;

218  v[4] = n5;

219  return v;

220  }

221  static std::vector<size\_t> to\_vec(size\_t n1, size\_t n2,

222  size\_t n3, size\_t n4,

223  size\_t n5, size\_t n6) {

224  std::vector<size\_t> v(6);

225  v[0] = n1;

226  v[1] = n2;

227  v[2] = n3;

228  v[3] = n4;

229  v[4] = n5;

230  v[5] = n6;

231  return v;

232  }

233  static std::vector<size\_t> to\_vec(size\_t n1, size\_t n2,

234  size\_t n3, size\_t n4,

235  size\_t n5, size\_t n6,

236  size\_t n7) {

237  std::vector<size\_t> v(7);

238  v[0] = n1;

239  v[1] = n2;

240  v[2] = n3;

241  v[3] = n4;

242  v[4] = n5;

243  v[5] = n6;

244  v[6] = n7;

245  return v;

246  }

247  static std::vector<size\_t> to\_vec(size\_t n1, size\_t n2,

248  size\_t n3, size\_t n4,

249  size\_t n5, size\_t n6,

250  size\_t n7, size\_t n8) {

251  std::vector<size\_t> v(8);

252  v[0] = n1;

253  v[1] = n2;

254  v[2] = n3;

255  v[3] = n4;

256  v[4] = n5;

257  v[5] = n6;

258  v[6] = n7;

259  v[7] = n8;

260  return v;

261  }

262  };

263

264

265  }

266

267 }

268

269 #endif

stan::io::var\_context::vals\_r

virtual std::vector< double > vals\_r(const std::string &name) const =0

Return the floating point values for the variable of the specified variable name in last-index-major ...

stan::io::var\_context::to\_vec

static std::vector< size\_t > to\_vec(size\_t n1, size\_t n2, size\_t n3, size\_t n4)

**Definition:** var\_context.hpp:201

stan::io::var\_context::to\_vec

static std::vector< size\_t > to\_vec(size\_t n1, size\_t n2, size\_t n3, size\_t n4, size\_t n5, size\_t n6)

**Definition:** var\_context.hpp:221

stan::io::var\_context::dims\_i

virtual std::vector< size\_t > dims\_i(const std::string &name) const =0

Return the dimensions of the specified floating point variable.

stan::io::var\_context::to\_vec

static std::vector< size\_t > to\_vec()

**Definition:** var\_context.hpp:179

stan::io::var\_context::validate\_dims

void validate\_dims(const std::string &stage, const std::string &name, const std::string &base\_type, const std::vector< size\_t > &dims\_declared) const

**Definition:** var\_context.hpp:124

stan::io::var\_context::to\_vec

static std::vector< size\_t > to\_vec(size\_t n1)

**Definition:** var\_context.hpp:182

stan::io::var\_context::dims\_r

virtual std::vector< size\_t > dims\_r(const std::string &name) const =0

Return the dimensions for the specified floating point variable.

stan::io::var\_context::to\_vec

static std::vector< size\_t > to\_vec(size\_t n1, size\_t n2, size\_t n3, size\_t n4, size\_t n5, size\_t n6, size\_t n7)

**Definition:** var\_context.hpp:233

stan::math::dims

void dims(const T &x, std::vector< int > &result)

**Definition:** dims.hpp:13

stan::io::var\_context

A var\_reader reads array variables of integer and floating point type by name and dimension...

**Definition:** var\_context.hpp:29

stan::io::var\_context::~var\_context

virtual ~var\_context()

**Definition:** var\_context.hpp:31

stan::io::var\_context::to\_vec

static std::vector< size\_t > to\_vec(size\_t n1, size\_t n2, size\_t n3)

**Definition:** var\_context.hpp:193

stan::io::var\_context::add\_vec

void add\_vec(std::stringstream &msg, const std::vector< size\_t > &dims) const

**Definition:** var\_context.hpp:114

stan::io::var\_context::contains\_i

virtual bool contains\_i(const std::string &name) const =0

Return true if the specified variable name has integer values.

stan::io::var\_context::contains\_r

virtual bool contains\_r(const std::string &name) const =0

Return true if the specified variable name is defined.

stan::io::var\_context::names\_r

virtual void names\_r(std::vector< std::string > &names) const =0

Return a list of the names of the floating point variables in the context.

stan::io::var\_context::to\_vec

static std::vector< size\_t > to\_vec(size\_t n1, size\_t n2)

**Definition:** var\_context.hpp:187

stan::io::var\_context::to\_vec

static std::vector< size\_t > to\_vec(size\_t n1, size\_t n2, size\_t n3, size\_t n4, size\_t n5, size\_t n6, size\_t n7, size\_t n8)

**Definition:** var\_context.hpp:247

stan::io::var\_context::vals\_i

virtual std::vector< int > vals\_i(const std::string &name) const =0

Return the integer values for the variable of the specified name in last-index-major order or the emp...

stan::io::var\_context::to\_vec

static std::vector< size\_t > to\_vec(size\_t n1, size\_t n2, size\_t n3, size\_t n4, size\_t n5)

**Definition:** var\_context.hpp:210

stan::io::var\_context::names\_i

virtual void names\_i(std::vector< std::string > &names) const =0

Return a list of the names of the integer variables in the context.


---

[ Stan Home Page ]

*© 2011–2014,
Stan Development Team.*
